# Transcriptomics and Metabolomics Reveal Purine and Phenylpropanoid Metabolism Response to Drought Stress in *Dendrobium sinense*, an Endemic Orchid Species in Hainan Island

**DOI:** 10.3389/fgene.2021.692702

**Published:** 2021-07-02

**Authors:** Cuili Zhang, Jinhui Chen, Weixia Huang, Xiqiang Song, Jun Niu

**Affiliations:** ^1^Key Laboratory of Genetics and Germplasm Innovation of Tropical Special Forest Trees and Ornamental Plants, Ministry of Education, College of Forestry, Hainan University, Haikou, Hainan, China; ^2^Engineering Research Center of Rare and Precious Tree Species in Hainan Province, College of Forestry, Hainan University, Haikou, Hainan, China

**Keywords:** *Dendrobium sinense*, drought stress, metabolomics, transcriptomics, third-generation sequencing, purine metabolism, phenylpropanoid biosynthesis

## Abstract

Drought stress is a bottleneck factor for plant growth and development, especially in epiphytic orchids that absorb moisture mainly from the air. Recent studies have suggested that there are complex transcriptional regulatory networks related to drought stress in *Dendrobium sinense.* In this study, the transcription and metabolite alterations involved in drought stress response in *D. sinense* were investigated through RNA-seq and metabolomics. A total of 856 metabolites were identified from stressed and control samples, with 391 metabolites showing significant differences. With PacBio and Illumina RNA sequencing, 72,969 genes were obtained with a mean length of 2,486 bp, and 622 differentially expressed genes (DEGs) were identified. Correlation analysis showed 7 differential genes, and 39 differential metabolites were involved in interaction networks. The network analysis of differential genes and metabolites suggested that the pathways of purine metabolism and phenylpropanoid biosynthesis may play an important role in drought response in *D. sinense*. These results provide new insights and reference data for culturally important medicinal plants and the protection of endangered orchids.

## Introduction

Through evolutionary processes, a series of response mechanisms has developed in plants that allow for chemical, physiological, and developmental responses to changes in the external environment (Claeys and Inze, 2013). Drought is one abiotic factor that strongly affects plant growth and development (Singh and Laxmi, 2015; Zhu, 2016). Several studies have elucidated important genes involved in drought stress response, mainly by studying gene expression patterns of drought-stressed plants (Joshi et al., 2016). For example, drought resistance in *Triticum aestivum* can be improved by *TaFBA* overexpression that affects the accumulation of sucrose and starch (Zhou et al., 2014). Additionally, aldehyde dehydrogenases (ALDHs) have been considered as general detoxifying enzymes that eliminate abiotic stress in a variety of organisms. Transgenic *Arabidopsis* plants expressing *CsALDH12A1* showed enhanced tolerance to drought stress during plant development (Duan et al., 2015). Under drought stress, the overexpressions of *allene oxide synthase* (*AOS*) and *allene oxide cyclase* (*AOC*) genes were positively correlated with the levels of major oxylipin metabolites in the *AOS* branch of the pathway, ultimately leading to the synthesis of jasmonates (Stenzel et al., 2003; Domenico et al., 2012). In CBL-interacting protein kinases (CIPKs) signaling, TaCIPK23 plays an important role in drought stress response in wheat (Cui et al., 2018).

Previous studies have reported that drought stress can induce many metabolites, such as carbohydrates and amino acids (Maruyama et al., 2009, 2014). Sugars serve not only as a solute to regulate cell osmotic pressure but also as primary messengers for signal transduction (Baena-González et al., 2007). Recent research in *Arabidopsis* indicated that glucose passes through hexokinase-dependent pathways and phytohormone response pathways, which has been proven to associate with participation in adversity (Sharma et al., 2019). *OsTF1L* overexpression in rice increased drought tolerance by regulating the gene expression involved in lignin biosynthesis (Bang et al., 2018). In addition, drought stress in rice seeds could induce the expression of *OsNCED3*, promoting the biosynthesis of abscisic acid (ABA) and inhibiting seed germination (Chen et al., 2019). Thus, abundant evidence shows that plants clearly regulate the accumulation of metabolites and gene transcription to respond to drought stress (Morimoto et al., 2017).

In recent years, Illumina sequencing has accelerated research of the transcriptome, which has made great achievements in functional gene verification (Du et al., 2016) and gene mining (Zhang et al., 2019). Although the short reads produced by this second-generation sequencing technology has high accuracy, the short-read length affects assembly and mapping accuracy in the absence of a reference genome, resulting in short transcript splicing and incomplete transcripts (Du and Sun, 2016). In contrast, third-generation long-read sequencing, such as PacBio, can generate full-length transcript sequences but has high error rates. Therefore, the depth of second-generation sequencing can complement the full-length transcript sequencing of third-generation sequencing, identifying subtypes with higher accuracy in transcriptome sequencing, especially for plants without reference genomes (Du and Sun, 2016).

*Dendrobium sinense* is an endangered epiphytic orchid plant with high medicinal value (Sun et al., 2014). It is well known that epiphytic orchids usually attach to smooth rocks and tree trunks that have low water retention capacity. Compared with terrestrial plants, epiphytes are subjected to moderate drought stress for most of the year (Zhang et al., 2016). As a consequence, moisture stress is the most important abiotic factor limiting the growth and development of epiphytic orchids (Zotz and Bader, 2009). However, few studies have combined the analysis of gene transcripts and metabolites to explore how epiphytic orchids will response to water deficit. In this present work, differential metabolites of *D. sinense* under drought stress were obtained by metabolome analysis. In addition to greatly increasing molecular resources devoted to *D. sinense* that lack genome sequence information, the combination of PacBio and Illumina sequencing illustrates gene expression patterns in drought stress. Combined with transcriptome and metabolome data, the importantly metabolic pathways related to drought response were identified in *D. sinense*. Our data provide new insights and reference data for improving drought resistance in these endangered orchids.

## Materials and Methods

### Plant Materials and Drought Stress Management

Wild *D. sinense* plants were collected at the national nature reserves of Bawangling, Changjiang County, Hainan Province, China. Individuals were cultured at room conditions [25°C, relative humidity (RH) ≥ 95%] for 2 months. Then, we simulated a range of drought conditions by adjusting relative air humidity (RH) in separate growing containers for 7 days. Based on our previous investigation (Yang, 2009), *D. sinense* was assigned to grow under conditions for group A (RH ≥ 95%), group B (45% ≤ RH ≤ 50%), or group C (RH ≤ 5%), which were named *D. sinense* A, B, and C (DSA, DSB, and DSC), respectively. After 7 days of growth in these conditions, leaves and pseudo-bulbs were collected for further metabolome and transcriptome studies, immediately frozen in liquid nitrogen and stored at –80°C.

For each treatment experiment, 100 mg sample was fully ground in liquid nitrogen for physiological test. The contents of catalase (CAT), peroxidase isoenzyme (POD), superoxide dismutase (SOD), and ascorbate peroxidase (APX) were determined using the respective Solarbio detection kits. The experimental data were analyzed by SPSS software.

### Metabolome Detection and Analysis

DSA, DSB, and DSC samples were crushed using a mixer mill (MM 400, Retsch, Shanghai, China) with a zirconia bead for 1.5 min at 30 Hz. One hundred milligrams powder was dissolved in 1.0 ml 70% aqueous methanol and incubated overnight at 4°C. Following centrifugation (10,000*g* for 10 min), the extracts were absorbed by CNWBOND Carbon-GCB SPE Cartridge (ANPEL, Shanghai, China) and filtered by SCAA-104 of 0.22 μm pore size (ANPEL, Shanghai, China). The data of high-performance liquid chromatography (HPLC) was analyzed by Shim-pack UFLC SHIMADZU CBM30A system (Applied Biosystems 6500 QTRAP, Thermo Fisher Scientific, Waltham, United States).

The extracted samples were analyzed using a liquid chromatography electrospray ionization tandem mass spectrometry (LC-ESI-MS/MS) system (UPLC, Shim-pack UFLC SHIMADZU CBM30A system; MS/MS, Applied Biosystems 6500 QTRAP). The conditions for liquid phase analysis were as follows: chromatographic column, Waters ACQUITY UPLC HSS T3 C18 (1.8 μm, 2.1 mm × 100 mm); solvent system, water (0.04% acetic acid)/acetonitrile (0.04% acetic acid); gradient program, 95:5 V/V at 0 min, 5:95 V/V at 11.0 min, 5:95 V/V at 12.0 min, 95:5 V/V at 12.1 min, 95:5 V/V at 15.0 min; flowrate, 0.40 ml/min; temperature, 40°C; and injection volume, 2 μl. The effluent was alternatively connected to an ESI-triple quadrupole-linear ion trap (QTRAP)-MS.

The ESI source operation parameters were as follows: electrospray ionization, source temperature 500°C; ion spray voltage (IS), 5,500 V; curtain gas (CUR) at 25.0 psi; and the collision gas (CAD), high. Instrument tuning and mass calibration were performed with 10 and 100 μmol/L polypropylene glycol solutions in QQQ and LIT modes, respectively. QQQ scans were acquired as multiple reaction monitoring (MRM) experiments with collision gas (nitrogen) set to 5 psi. Declustering potential (DP) and collision energy (CE) for individual MRM transitions were done with further DP and CE optimization. A specific set of MRM transitions were monitored for each period according to the metabolites eluted within this period (Chen et al., 2013).

### Metabolome Data

The software *analyst 1.6.3* was used to process the mass spectrometry data, and the metabolites of the samples were qualitatively and quantitatively analyzed based on Met Ware metabolic database (Wuhan, China). Orthogonal partial least-squares discriminant analysis (OPLS-DA) with supervised mode was used to analyze the differences within and among experimental groups. The metabolic contents were normalized by R software^1^, through which an accumulation-mode clustering analysis [hierarchical clustering analysis (HCA)] of metabolites was performed to test for differences among samples (Chong and Xia, 2018). The pathway-based Kyoto Encyclopedia of Genes and Genomes (KEGG) database was used for the functional annotation of differential metabolites (Kanehisa and Goto, 2000).

### RNA Isolation and RNA-Seq Library Construction

Total RNA was extracted from mature non-aging leaves and pseudo-bulbs with the Quick RNA Isolation Kit (Waryong, Beijing, China). RNA quality and concentration of each sample were measured using agarose gel electrophoresis, Nanodrop 2000 (Thermo Fisher Scientific, Waltham, United States), and Agilent Bioanalyzer 2100 (Agilent Technologies, Santa Clara, United States). Subsequently, an Iso-Seq library was prepared using the Clontech SMARTer PCR cDNA Synthesis Kit and sequenced on a PacBio Sequel (PN 100-092-800-03).

### Transcriptome Data Analysis

SMRTlink 6.0 software was used to process raw sequence data, and circular consensus sequences (CCS) were obtained by subread BAM files. The CCS BAM files were then classified as full-length non-chimera (FLNC) or non-full length (nFL) according to 5′-primer, 3′-primer and poly-A. Consensus sequences were obtained by clustering full-length sequences with isoform-level clustering (ICE), and additional nucleotide errors in consensus reads were corrected using the Illumina RNA-seq data using the LoRDEC software (Salmela and Rivals, 2014). The consensus sequences were corrected with non-full-length and non-chimeric sequences, resulting in polished consensus sequences (Fu et al., 2012). Finally, any redundancy in corrected consensus reads was removed by CD-HIT to obtain transcripts for subsequent analyses and as reference sequences (Fu et al., 2012). The RSEM software was used to estimate gene expression levels for each sample by mapping the transcript sequences to obtain the read count of each transcript (Li and Dewey, 2011). Considering the sequence depth and gene length of fragments, the gene expression level was calculated with the fragments per kilobase million (FPKM) method.

### The Analysis of Differential Expression and Functional Annotation

DESeq-R package (1.10.1) was used to analyze the differential expression between two groups. The differentially expressed genes (DEGs) were selected by | log2 (fold change)| > 1 and false discovery rate (FDR) < 0.05 (Storey and Tibshirani, 2003). Gene annotations and gene ontology (GO) enrichment analyses were implemented with the Go seq-R package. GO terms with FDR ≤ 0.05 were considered significantly enriched by DEGs. The KEGG database was used as a resource for understanding high-level functions and utilities of the biological system^2^. The KOBAS (3.0) software was used to test the statistical enrichment of DEGs in KEGG pathways.

### Statistical Analysis

The Pearson coefficient between metabolome and transcriptome data were calculated using R (cor test) software. Correlations corresponding to *R*^2^ > 0.8 and FDR < 0.05 were selected. The correlated data were visualized using Cytoscape software and KEGG database. Differences between samples were tested for statistical significance using the Duncan MRT method. Statistical analysis was implemented by SPSS software (version 19.0).

## Results

### Effects of Drought Stress on Protective Enzymes in Pseudobulbs of *D. sinense*

**FIGURE 1:**
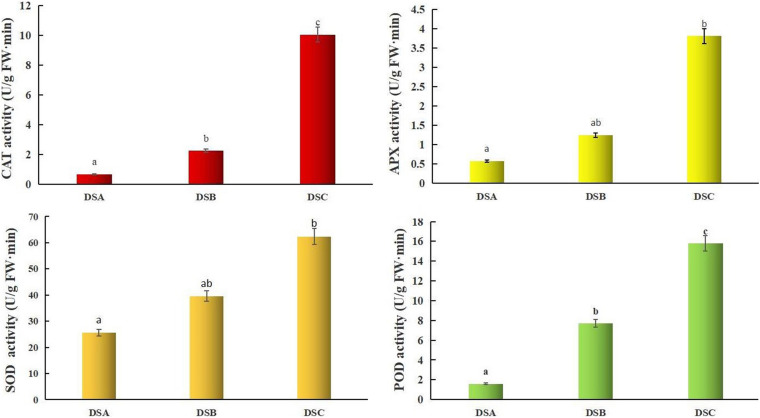
Effects of drought stress on physiological and biochemical parameters in pseudobulb of *D. sinense*. Data are means ± SE of three separate measurements. The significance of differences was analyzed using *Duncan* method.

In order to explore the physiological mechanism of *D. sinense* response to drought stress, three treated experiments were conducted with different RH gradients sustained for 7 days. The color of DSA leaves was healthy green, while DSB and DSC leaves showed a noticeably yellow color with the increase in stress degree (Supplementary Figure 1). There are protective enzymes to scavenging reactive oxygen species (ROS) in plants, and the high activity of the enzyme is closely related to the degree of stress. Catalase and POD activities displayed a significant gradual increase (*p* < 0.05) with the intensification of drought stress (Figure 1). Superoxide dismutase and APX protective enzyme activities were only significantly different (*p* < 0.05) between DSA and DSC (Figure 1). The results showed that the activity of protective enzymes in *D. sinense* was increased, and the scavenging ability of reactive oxygen species was enhanced after drought stress.

### Identification and Annotation of Metabolites in *D.sinense*

**FIGURE 2:**
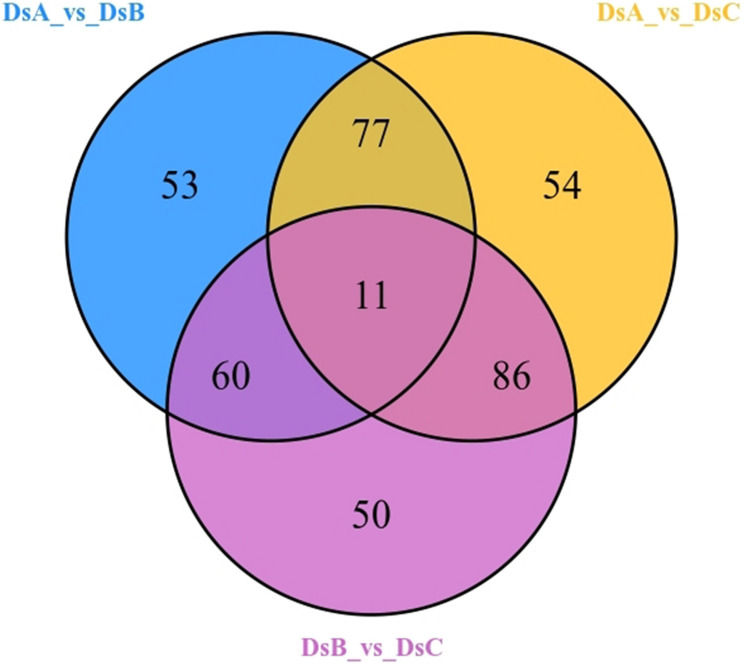
Differential metabolites by Venn diagram.

A total of 856 metabolites were detected, belonging to 16 categories (Supplementary Table 1). Among them, the most abundant were flavonoids (240; 28.04%), organic acids and derivatives (106; 12.38%), and amino acids and derivatives (93; 10.86%). Differential abundance of metabolites indicated 391 differential metabolites related to drought stress (Figure 2), mainly including flavonoids (116, 29.67%), phenylpropanoids (38, 9.72%), amino acids and derivatives (37, 9.46%), and lipids (35, 8.95%) (Supplementary Table 1). Interestingly, upregulated metabolites were more common than downregulated metabolites under drought stress, as observed in the comparisons of DSA vs. DSB and DSA vs. DSC groups (Supplementary Figure 2). Additionally, when comparing DSA (control) to DSB (moderate drought stress), 32 of the 151 identified upregulated metabolites were increased by more than 2.5-fold with a variable importance in project (VIP) score > 1.5. Of these, acetosyringone, 4-methylcatechol, pyrocatechol, apigenin 6-C-hexosyl-8-C-hexosyl-O-hexoside, and parthenolide increased most dramatically by more than >2,000-fold change and VIP > 1.7 (Supplementary Table 2). DSA vs. DSC (extreme drought stress) also included 32 upregulated metabolites with more than 2.5-fold change and VIP > 1.5. Of these, c-hexosyl-apigenin c-pentoside, acetosyringone, magnolol, 4-methylcatechol, pyrocatechol, and catechol increased by more than 2,000-fold (Supplementary Table 2). Intriguingly, 13 of the same metabolites with significant differences were detected in DSA vs. DSB and DSA vs. DSC, such as, L-tryptamine, S-(5′-adenosy)-L-homocysteine, *p*-coumaric acid, and 4-methylcatechol (Supplementary Table 2).

**FIGURE 3:**
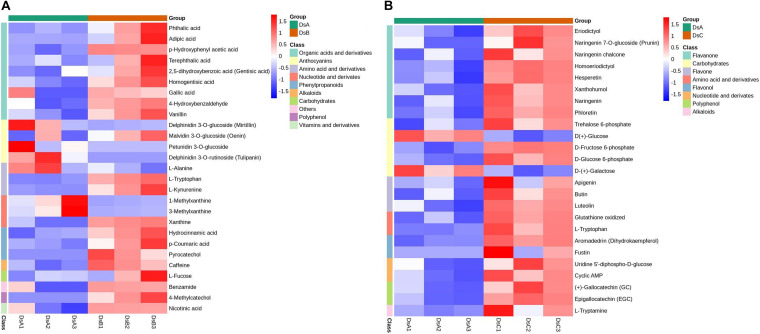
Metabolites on metabolic pathways with significantly different enrichment. **(A)** Cluster analysis of DSB vs. DSA differential metabolites. **(B)** Cluster analysis of DSC vs. DSA differential metabolites. The analysis of hierarchical clustering for differential metabolism calculated by complete linkage approach. These metabolites were derived from metabolic pathways (*p* < 0.05) in DSA vs. DSB and DSA vs. DSC. Red denotes metabolites with high content, and blue means low content. The agglomeration method to be used by hclust function.

To further study the response to drought stress, KEGG enrichment pathway analysis was performed (Supplementary Table 3). The differential metabolites in the significant enrichment pathway were analyzed by cluster analysis. Under moderate drought stress, organic acids and their derivatives and procyanidin metabolites were significantly enriched. Under more extreme drought stress (RH ≤ 5%), flavonoids and carbohydrates were significantly enriched (Figure 3 and Supplementary Table 3).

### Transcriptome Sequencing of *D. sinense*

To obtain a more reliable transcriptome library, different treatments and organs of *D. sinense* were prepared for third-generation sequencing. 18.41G polymerase read bases (504,082 polymerase read) were obtained on the PacBio Sequel platform. Additionally, 84.47G clean bases were obtained on an Illumina platform (Supplementary Table 4). A total of 72,797 unigenes with a mean length of 2,486 bp were obtained (Table 1). We found that 62,038 (85.02%) unigenes had been previously annotated at least in one database (Supplementary Figure 3). Through GO analysis, we categorized these unigenes into 56 pathways, with “metabolic process,” “cell,” and “catalytic activity” having high ranking in biological processes, cellular components, and molecular functions, respectively (Supplementary Figure 3). A total of 38,037 unigenes were divided into 25 groups using the KOG database. “General function prediction only” was the most significant category, follow by “Posttranslational modification protein turnover chaperones” and “Signal transduction mechanisms” (Supplementary Figure 3).

**TABLE 1:** Summary of non-redundant transcriptional sequences.

Polymerase read bases (G)	Polymerase reads	CCS number	Mean length	Consensus number	Mean length	Unigene number	Mean length
18.41	504,082	424,187	1,955	140,872	2,244	72,969	2,486

### Drought Inducible DEGs in *D. sinense*

**FIGURE 4:**
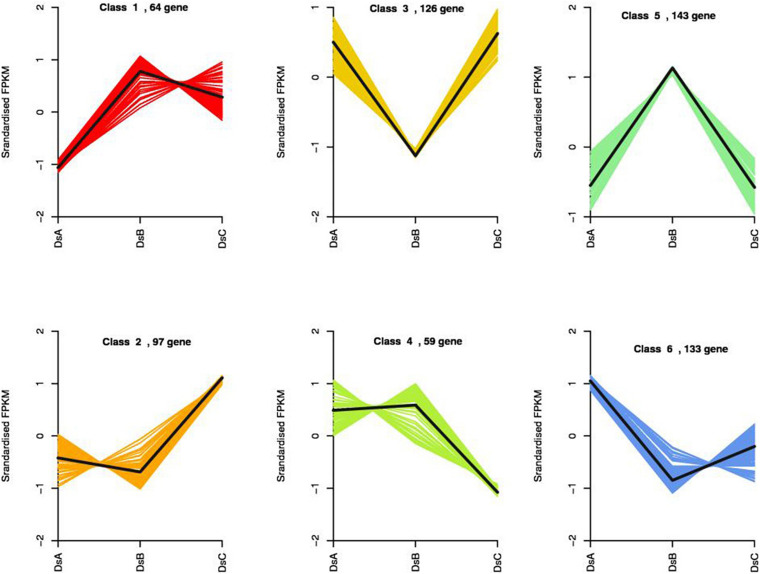
Results of the k-means clustering of 622 DEGs identified by PacBio Iso-Seq based on their expression patterns.

We compared transcript counts across treatments to identify DEGs. Three hundred seventy-four DEGs were identified in DSA vs. DSB (151 upregulated and 223 downregulated genes), 130 DEGs in DSA vs. DSC (53 upregulated and 77 downregulated genes), and 256 DEGs in DSB vs. DSC (129 upregulated and 127 downregulated genes) (Supplementary Figure 4). Statistically, a total of 622 DEGs were characterized in differently treated *D. sinense* (Supplementary Figure 4). Surprisingly, DEGs were not found to be shared among different comparison groups (Supplementary Figure 4). To further explore the expression patterns of DEGs in response to drought stress, 622 DEGs were grouped into six clusters by k-means (Figure 4). Compared with control (DSA), the DEGs from class 1 showed a positive response, while those of class 6 showed a negative response (Figure 4). In DSC, the class 2 DEGS were upregulated, and the class 4 DEGs were downregulated (Figure 4). On the other hand, in DSB, the class 3 DEGs were upregulated, but the class 5 DEGs were downregulated (Figure 4). In the GO analysis, the functions of class 1 were related to “carbohydrate derivative binding,” “adenyl nucleotide binding,” “ATPase activity,” and “nucleotide binding” (Supplementary Table 5). “Transcription factor activity,” “oxidoreductase activity,” and “pectin metabolic process” were identified from class 2 of DEGs (Supplementary Table 5).

### Identification of Transcription Factors in Candidate Genes

**FIGURE 5:**
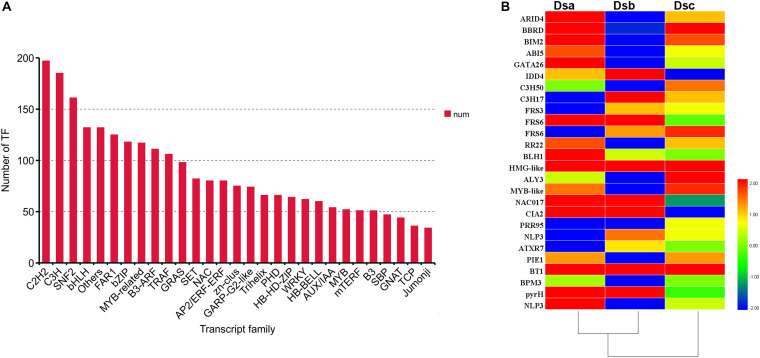
TFs involved in response to drought stress. **(A)** Number of TFs in different families. **(B)** Heat map presenting the expression patterns of different TFs in response to drought treatments. Red denotes TFs with high expression levels, and blue means low expression. The color ranges from red to blue represents the log_2_ (FPKM + 0.01) value from large too small.

Transcription factors (TFs) are involved in regulating the biosynthesis of secondary metabolites in plants. According to the plant transcription family database, 3,284 TFs were identified in our data and distributed across 89 families using iTAK software (Figure 5A). Importantly, 27 differentially expressed TFs, classified into 21 families, were identified (Figure 5B). When combined with the analysis of expression trends of DEGs, the TFs were distributed across different classes. Class 3 contained the most differentially expressed TFs (ARID4, BIM2, C3H50, RR22, MYB-like, BT1, and BPM3). Class 5 contained the second most differentially expressed TFs (IDD4, C3H17, FRS6, HMG-like, PIE1, and pyrH). Class 6 consisted of five differentially expressed TFs (BBRD, ABI5, GATA26, BLH1, and NLP3). The ABI5 TF is involved in ABA signal transduction through a negative regulatory relationship. Classes 1 and 4 have the same number of differentially expressed TFs: FRS3, ATXR7, and PIE1 from class 1 and FRS6, NAC017, and CIA2 from class 4. Additionally, the TFs of PRR95 and ALY3 were upregulated after extreme drought stress (RH ≤ 5%). These results suggested that TFs may regulate the metabolite biosynthesis pathway of *D. sinense* in response to drought stress.

### Coexpression Networks of *D. sinense* in Response to Drought Stress

We then explored correlations between the DEG and metabolites by calculating the Pearson correlation coefficients between all DEGs and differential metabolites. One hundred twenty-one DEGs and 239 differential metabolites had a strong correlation (*R*^2^ > 0.8, FDR < 0.05) (Supplementary Table 6). We then screened for extremely significantly DEGs (*p* < 0.01), which resulted in a set of DEGs that were enriched for genes relating to redox process, metabolic process, and phospholipid biosynthesis process (Supplementary Table 7). Nine extremely significantly DEGs were involved in microtubule organizing center and nuclear cell components, and 24 extremely significantly DEGs related to molecular functions were involved in molecular processes such as 6-phosphofructokinase activity, methionine adenosyltransferase, and GTP binding (Supplementary Table 7).

**FIGURE 6:**
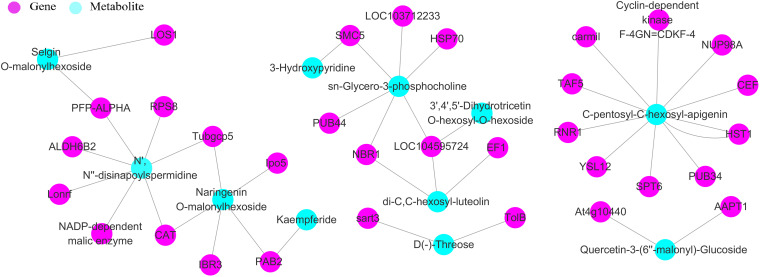
Coexpression network of DEGs and differentially expressed metabolites. These DEGs were correlated with metabolites (*R* > 0.8). The networks were visualized with the Cytoscape software. The purple denotes represent DEGs, and blue means differential metabolites.

We also constructed a coexpression network of 33 extremely significantly DEGs and 11 differential metabolites (Figure 6). The result showed that extremely significant DEGs may play an important role in the response of *D. sinense* to drought stress. Further analysis on the relationship between genes and metabolites found five metabolic pathways in the coexpression network, such as glycerophospholipid metabolism pathway (ko0564), arginine and proline metabolism pathway (ko0330), purine metabolism pathway (ko0230), tryptophan metabolism pathway (ko0380), and phenylpropanoid biosynthesis pathway (ko0940).

### Purine Metabolism and Phenylpropanol Biosynthesis of *D. sinense* in Response to Drought

**FIGURE 7:**
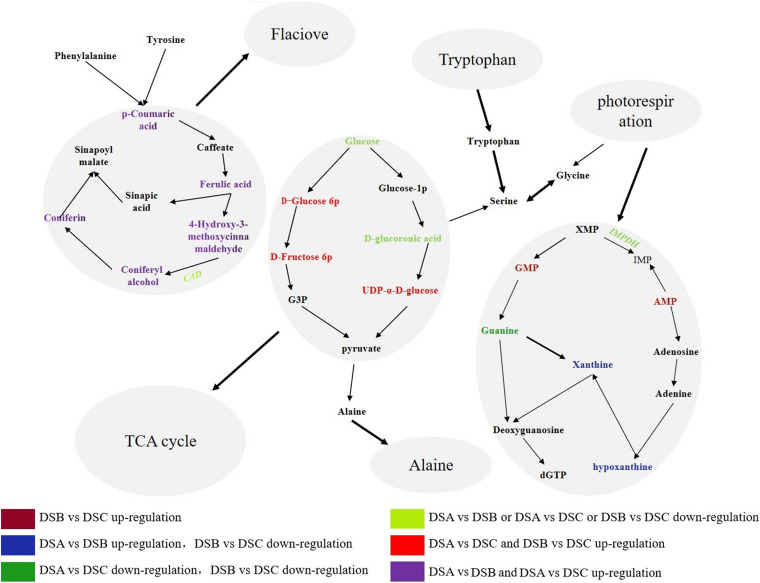
Pathway diagram of major metabolic pathways. This pathway is constructed based on the KEGG pathway. The color denotes the different expression levels. Dark red denotes upregulation in DSB vs. DSC, blue shows that DEGs or metabolites are upregulated in DSA vs. DSB and downregulation in DSB vs. DSC, strong green denotes downregulation in DSA vs. DSB and DSB vs. DSC. Blue denotes downregulation in DSA vs. DSB or DSA vs. DSC or DSB vs. DSC, red denotes upregulation in DSA vs. DSB and DSB vs. DSC, and purple denotes with upregulation in DSA vs. DSC and DSB vs. DSC.

By combining the correlation analysis of transcriptome and metabolome in *D. sinense*, we found that purine metabolism and phenylpropanol biosynthesis were the main metabolic pathways in response to drought stress. The analysis found 7 DEGs and 39 differential metabolites from the purine metabolic pathway, such as *phosphatidylglycamine synthase* (*purL*), *adenylate kinase* (*AK*), and *ribonucleoside diphosphate reductase subunit M1* (*RRM1*). In addition, the expression of *inosine-5′-monophosphate dehydrogenase* (*IMPDH*) gene was upregulated specifically in DSB (the moderate drought conditions) (Figure 7). Compared with DSA, the metabolite accumulation of 3,4-dihydroxy-DL-phenylalanine in DSB and DSC was downregulated, and the contents of guanine, xanthine, and hypoxanthine were upregulated. For example, xanthines accumulated significantly in DSB (DSB > DSA > DSC). Therefore, the biomass accumulation of xanthine, hypoxanthine, and guanine was consistent with the expression trend of *IMPDH* gene under drought stress. In addition, five *PAL*, four *4CL*, seven *CAD*, two *CCR*, and two *cytochrome P450* genes were found in the phenylpropanoid biosynthesis pathway. Compared with DSA, these genes did not show significant differences in DSB and DSC. However, the *CAD* gene was downregulated in DSB vs. DSC. Surprisingly, the phenylpropanol biosynthetic pathway of downstream metabolites [coumaric acid, conibenol, (E)-p-coumaric acid, and 4-hydroxy-3-methoxy cinnamaldehyde] was upregulated compared with DSA. In addition, the glycolysis pathway was also involved in response to drought stress (Figure 7). These results indicate that *D. sinense* improves its ability to resist drought stress by regulating the metabolism of genetic material and promoting the accumulation of antioxidant products and cytoplasmic material under drought stress.

## Discussion

### Third-Generation Sequencing Reveals the Transcriptome Complexity in *D. sinense*

Compared to genomic DNA-seq, RNA-seq is often faster, simpler, and more scalable and can reveal important information about gene transcription and expression (Trapnell et al., 2010; José and Marta, 2014). Extensive transcriptomic studies have shown that RNA-seq technology is a useful tool for studying adaption of plants to different environmental stress (Brenner and Schmülling, 2012; José and Marta, 2014; Begara-Morales et al., 2014). However, many studies had found that second-generation RNA-seq technology is imperfect, and short reads can reduce sequence and assembly accuracy (Haas and Zody, 2010; Grabherr et al., 2013). With the development of newer sequencing technologies, full-length transcript sequences can be obtained using the PacBio Iso-Seq technology. This technology solves the inherent limitations of Illumina high-throughput sequencing and identifies new gene subtypes with alternatively spliced transcript variants (Roberts et al., 2013; Du and Sun, 2016; An et al., 2018). Previous Illumina-based RNA-seq studies in orchids found the average transcript length to be 764.3 bp in *Dendrobium huoshanense* (Yuan et al., 2018) and 505 bp in *Dendrobium officinale* (He et al., 2017). In this present work, the full-length transcripts had an average length of 2,244 bp, similar to previous results of *Dendrobium candidum* (Xu et al., 2017). These results support the viewpoint that the third-generation sequencing technique can obtain longer transcripts than Illumina-based RNA-seq. To obtain a complete and accurate transcriptome library, short-reads generated from Illumina sequencing were used to correct long reads from third-generation sequencing. Previous studies have used this strategy to generate high-throughput sequencing data, providing reference transcriptomes for plant species that lack genome sequence information (Sharon et al., 2013; Grabherr et al., 2013; Huddleston et al., 2014). To our knowledge, this is the first report of transcriptome sequencing using RNA-seq and PacBio SMRT in *D. sinense*. This full-length reference transcriptome will be helpful to future genetic studies of *D. sinense*.

### Metabolic Responses to Drought Stress in *D. sinense*

It is well known that multiple adaptative mechanisms have evolved in plants to reduce the effects of stress. Metabolites appear to be the key to these adaptations, with many studies revealing significant differences in the accumulation of metabolites under drought stress (Georgii et al., 2017; Navarro-Cerrillo et al., 2019). In this study, flavonoids, amino acids and derivatives, organic acids and derivatives, and phenylpropanoids were identified as accumulating at significantly different rates across drought conditions, showing upregulation under drought stress in both DSA vs. DSB and DSA vs. DSC. These results are similar to previous studies that also found that the content of flavonoids, amino acids, and other metabolites were increased in drought stress (Georgii et al., 2017; Navarro-Cerrillo et al., 2019). A previous study in *Dendrobium moniliforme* also indicated that the accumulation of flavonoids and alkaloid was induced by drought stress (Wu et al., 2016). Physiologically, these secondary metabolites in plants can prevent the accumulation of reactive oxygen species from damaging proteins and cell membranes, thereby improving drought resistance (Mutwakil et al., 2017; Šircelj et al., 2017). Specifically, organic acids and small molecule compounds may be involved in carbon metabolism (López-Bucio et al., 2000). From a physiological and biochemical perspective, the increase in flavonoid content in the pseudobulbs of *D. sinense* appears to be related to adaptability to water (Zhu, 2010; Šircelj et al., 2017). The extremely significant accumulations of amino acids, organic acids, and derivatives in drought-stressed *D. sinense* further suggest that these are important metabolites in response to drought stress (López-Bucio et al., 2000). In addition, only a few secondary metabolites showed a strong response to drought stress, such as four phenylpropanoids, two flavone, one polyphenol, and one parthenolide. Importantly, acyl syringone, 4-methylcatechol, catechol, apigenin6-C-hexosyl-8-C-hexosyl-O-hexoside, parthenolide, magnolol, and pyrocatechol may be key molecular components of drought stress response in *D. sinense*. Importantly, strong drought tolerance in *D. sinense* may in fact be related to its high flavonoid content in the body. This strongly suggests that drought stress induces the accumulation of metabolites that may improve drought tolerance of *D. sinense*.

### Functional Clustering of DEGs and Metabolites Response to Drought Stress in *D. sinense*

Previous work has shown that plants adapt to drought stress by responding to the accumulation of metabolites and altering gene expression (Georgii et al., 2017; Acevedo et al., 2019). Although many drought-resistant genes have been found in model plants, few investigations have been done on *Dendrobium*. Our study presents a systematic analysis of drought-resistance genes, shedding light on the mechanisms of drought response in *Dendrobium*. In *D. sinense*, 622 DEGs were identified as potentially involved in drought stress. Through GO and KEGG enrichment analyses, we found that these DEGs were significantly enriched in ATPase activity, hydrolase activity, acting on acid anhydrides, pyrophosphatase activity, ion binding, and RNA helicase activity. Moreover, many DEGs encoding protein kinases, lipoxygenase, phospholipase, antioxidant enzymes, transporters, ubiquitin-protein ligase, and TFs were identified, such as *FBA1*, *CIPK32*, *PBL27*, *LOX2*, *PLC4*, *GAD*, *ABCF1*, *KEG*, *GATA26*, and *C3H*. Notably, *LOX2* and *KEG* are involved in the biosynthesis and signal transduction of plant hormones (Wasternack, 2007; Wasternack and Hause, 2013, 2019). *ABI5* and *EGS* are related to ABA biosynthesis (Joshi et al., 2016; Zhu, 2016). These results clearly suggest that hormone signaling may play an important role in the response of *D. sinense* to drought stress.

### Underlying the Responses of Phenylpropanoid Biosynthesis and Purine Metabolism in *D. sinense* to Drought Stress

Studies in model plants have revealed that nucleotide biosynthesis and degradation are two of the metabolic pathways involved in drought response in *Arabidopsis* (Watanabe et al., 2014) and rice (Boo and Jung, 1998). Recent work indicated that the nucleotide metabolism pathway was an important pathway for *D. wangliangii* to adapt to drought stress (Zhao et al., 2019). The *IMPHD* gene was reported as the rate-limiting enzyme of guanine nucleoside biosynthesis, directly affecting the biosynthesis of guanine, xanthine, and hypoxanthine (Mercati et al., 2019). In DSA vs. DSB, the *IMPHD* gene was upregulated, and the content of guanine, xanthine, and hypoxanthine increased. In DSB vs. DSC, the expression level of *IMPHD* gene was downregulated and the metabolites guanine, xanthine, and hypoxanthine were also downregulated. These data indicated that drought stress affected the biosynthesis of purines pathway.

Previous work showed that when guanine is deficient, guanine is replaced by xanthine and hypoxanthine in RNA and DNA synthesis, leading to disorders of the metabolic system (Zoref-Shani et al., 2010). Our results showed that guanine biosynthesis was promoted in response to drought stress in *D. sinense*, while xanthine and hypoxanthine bioaccumulation was reduced under severe drought stress. This allowed for the maintenance of normal metabolic processes to reduce stress-induced damage. In addition, previous studies have reported that nucleotide metabolism may increase drought tolerance in *Hylocereus undatus* (Fan et al., 2014) and *Triticum boeoticum* (Liu et al., 2015). This suggested that purine metabolism may increased the drought tolerance of *D. sinense*. This result therefore supports findings from previous studies and provides a new insight to explore drought tolerance of epiphytes.

*PAL*, *C4H*, and *4CL* are key genes in the early stages of lignin and flavonoid biosynthesis (Martens and Mithöfer, 2005). In *Fagopyrum esculentum* Moench, lignin increases cell wall thickness to prevent water loss and improve drought resistance (Hou et al., 2019). In our study, we found that coumaric acid, coniferol, (E)-p-coumaric acid, and 4-hydroxy-3-methoxy cinnamaldehyde were upregulated in DSB vs. DSC, and five genes related to lignin were also identified. For these genes, only the *CAD* gene was downregulated in DSA vs. DSB, and no other genes were significantly different in DSB vs. DSC. This negative correlation between lignin biosynthesis and gene expression under drought stress is consistent with results from previous studies (Hu, 2008; Liu, 2019). Reports have also suggested that the root tissue thickness of *D. sinense* roots increased under drought stress (Wang, 2017). Our results suggest that when *D. sinense* experience drought stress, lignin accumulation is promoted, thereby increasing cell wall thickness to prevent water loss. In addition, most flavonoids were significantly upregulated in DSB vs. DSC even though we did not identify DEGs associated with flavonoid biosynthesis. However, previous research has shown that genes related to flavonoid biosynthesis were upregulated in buckwheat under drought stress (Hou et al., 2019). It is also suspected that flavonoids have antioxidant properties (Sarker and Oba, 2018). In light of this, our results suggest that *D. sinense* responds to water deficit in the air by increasing its antioxidant capacity, but the pathway for regulating flavonoid biosynthesis under drought stress needs further exploration.

## Data Availability Statement

The datasets presented in this study can be found in online repositories. The names of the repository/repositories and accession number(s) can be found below: NCBI BioProject Accession PRJNA723915.

## Author Contributions

XS, JN, and CZ conceived the project and designed the experiments. CZ, WH, and JC collected the datasets. CZ and JC plotted the figures. CZ, JC, XS, and JN wrote the manuscript. All authors read and approved the final manuscript.

## Conflict of Interest

The authors declare that the research was conducted in the absence of any commercial or financial relationships that could be construed as a potential conflict of interest.

## Footnotes

1 www.r-project.org/

2 http://www.genome.jp/kegg/

## Supplementary Material

Supplementary Figure 1 The phenotypes of *D. sinense* under drought stress. **(A,D)** were growth in RH ≥ 95%, **(B,E)** were growth in 45% ≤ RH ≤ 50%, **(C,F)** were growth in RH ≤ 5%

Supplementary Figure 2 Different metabolites up- or down-regulated in different groups. **(A)** DSA vs. DSB, **(B)** DSA vs. DSC, **(C)** DSB vs. DSC.

Supplementary Figure 3 Functional prediction and annotation of transcriptome genes in *D. sinense. (A) D. sinense* GO enrichment function analysis. The abscissa is the GO term of the next level in the three major categories of GO, and the ordinate is the number of genes annotated to the term (including its sub-term). The three different classifications represent the three basic classifications of GO term from left to right (i.e., biological processes, cell components, and molecular functions). **(B)** *D. sinense* gene function prediction. The abscis-axis is the names of the 26 groups of KOG, and the ordinate is the number of genes annotated to the group. **(C)** *D. sinense* gene functional annotation Venn diagram. The sum of the numbers in each large circle represents the number of transcripts for the database annotation, and the overlapping parts of the circles represent the transcript annotation results that are common between databases.

Supplementary Figure 4 Number and distribution of up-regulated and down-regulated genes. **(A)** The distribution of DEGs in different sample, **(B)** Summary of *D. sinense* DEGs under drought stress.

Supplementary Table 1 Detection of all metabolic quantities and species.

Supplementary Table 2 Significantly different metabolites between stressed and control samples.

Supplementary Table 3 Significantly enriched KEGG pathways.

Supplementary Table 4 Summary of RNA-seq for DSA, DSB, and DSC.

Supplementary Table 5 GO enrichment result in DEGs.

Supplementary Table 6 Differential genes and differential metabolites with a correlation coefficient *R*^2^ ≥ 0.8.

Supplementary Table 7 Extremely significantly DEGs GO function annotation.
