# Supplementary figures and images for: Transcriptomics and Metabolomics Reveal Purine and Phenylpropanoid Metabolism Response to Drought Stress in Dendrobium sinense, an Endemic Orchid Species in Hainan Island
Source: Front Genet. 2021 Jul 2;12:692702. doi: 10.3389/fgene.2021.692702 (PMC8283770; doi:10.3389/fgene.2021.692702)

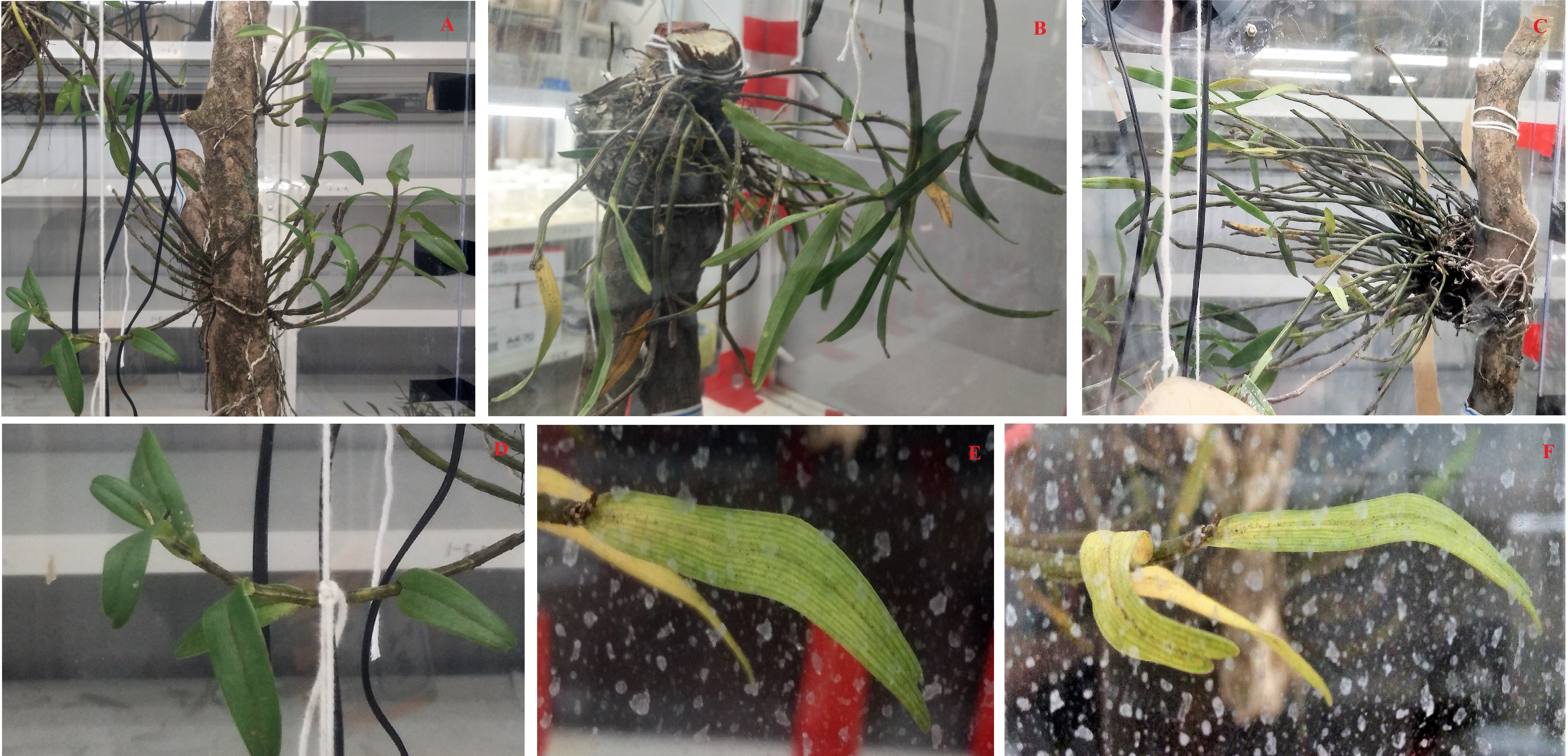

Supplement: Supplementary Figure 1 — The phenotypes of D. sinense under drought stress. (A,D) were growth in RH ≥ 95%, (B,E) were growth in 45% ≤ RH ≤ 50%, (C,F) were growth in RH ≤ 5% [file Image_1.JPEG]

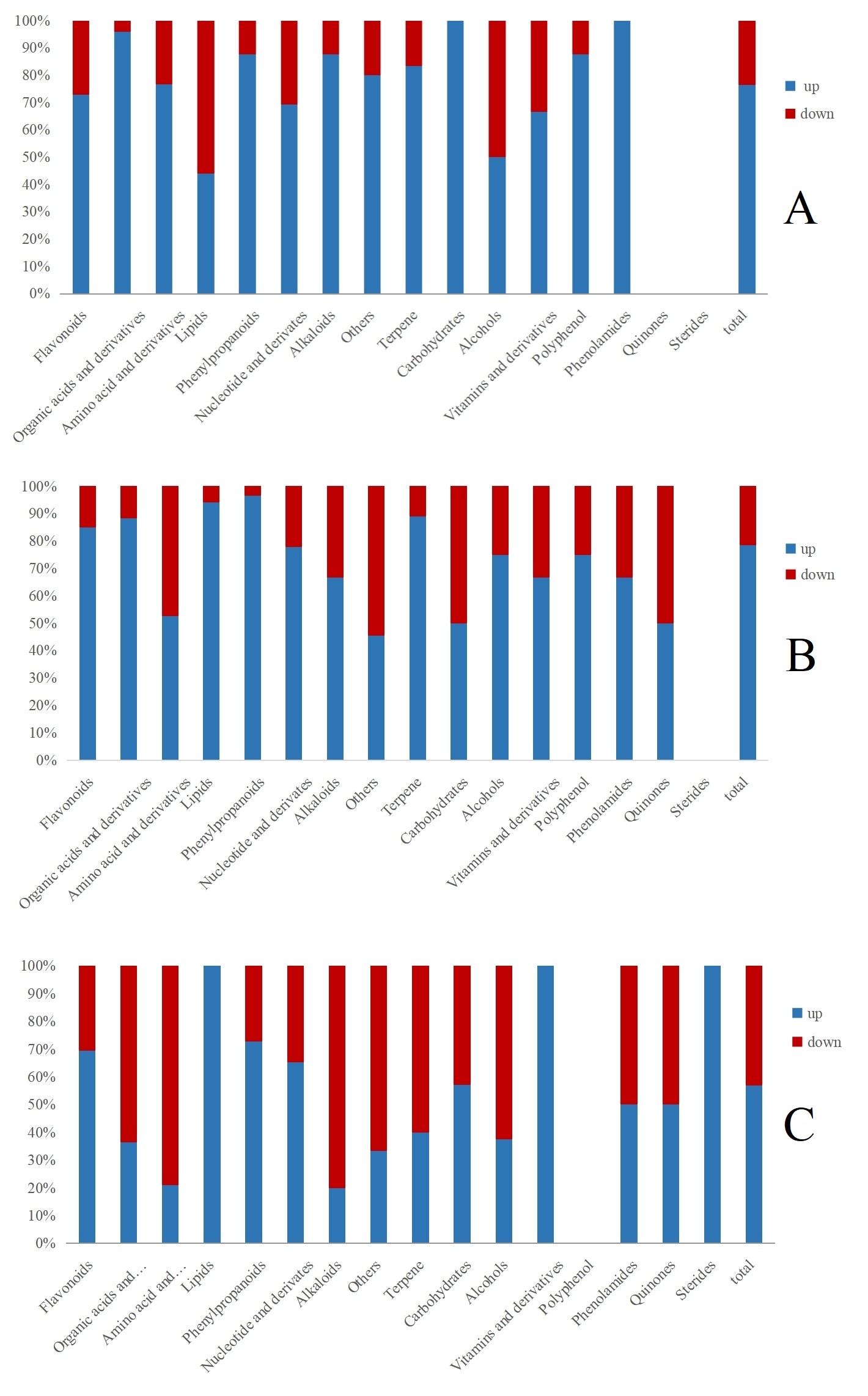

Supplement: Supplementary Figure 2 — Different metabolites up- or down-regulated in different groups. (A) DSA vs. DSB, (B) DSA vs. DSC, (C) DSB vs. DSC. [file Image_2.JPEG]

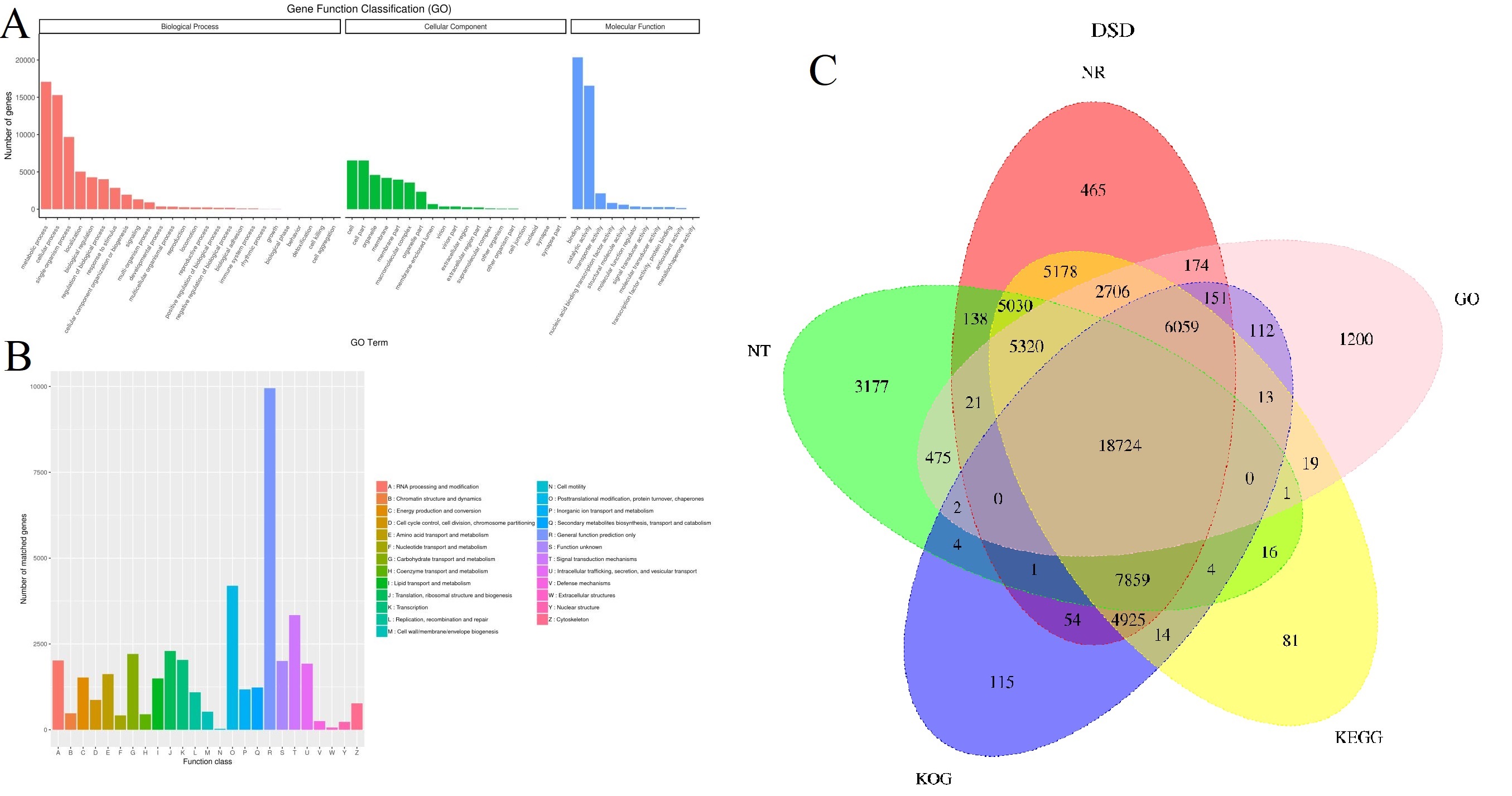

Supplement: Supplementary Figure 3 — Functional prediction and annotation of transcriptome genes in D. sinense. (A) D. sinense GO enrichment function analysis. The abscissa is the GO term of the next level in the three major categories of GO, and the ordinate is the number of genes annotated to the term (including its sub-term). The three different classifications represent the three basic classifications of GO term from left to right (i.e., biological processes, cell components, and molecular functions). (B) D. sinense gene function prediction. The abscis-axis is the names of the 26 groups of KOG, and the ordinate is the number of genes annotated to the group. (C) D. sinense gene functional annotation Venn diagram. The sum of the numbers in each large circle represents the number of transcripts for the database annotation, and the overlapping parts of the circles represent the transcript annotation results that are common between databases. [file Image_3.JPEG]

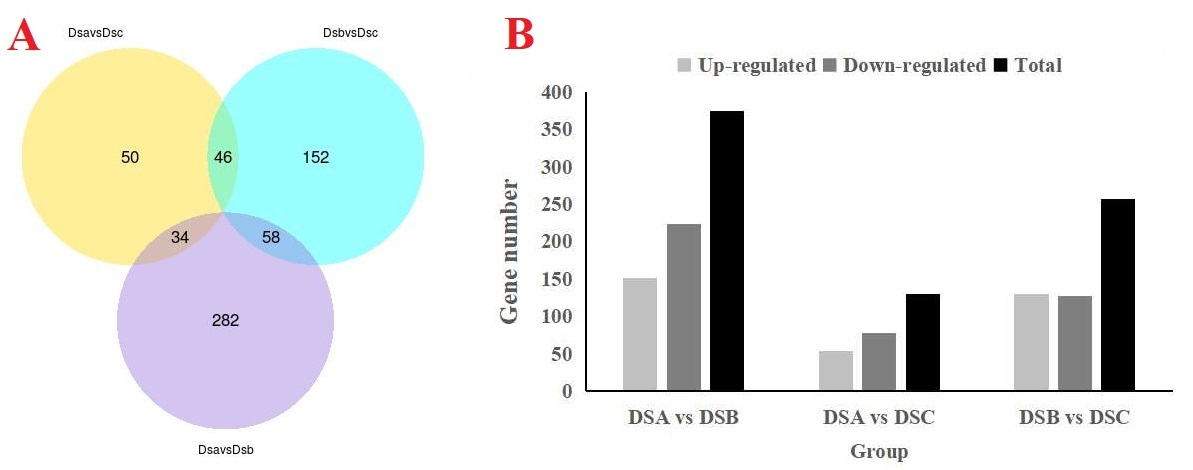

Supplement: Supplementary Figure 4 — Number and distribution of up-regulated and down-regulated genes. (A) The distribution of DEGs in different sample, (B) Summary of D. sinense DEGs under drought stress. [file Image_4.JPEG]
