# Supplementary material for: Transcriptomics and Metabolomics Reveal Purine and Phenylpropanoid Metabolism Response to Drought Stress in Dendrobium sinense, an Endemic Orchid Species in Hainan Island
Source: Front Genet. 2021 Jul 2;12:692702. doi: 10.3389/fgene.2021.692702 (PMC8283770; doi:10.3389/fgene.2021.692702)
Supplement: Supplementary Table 4 — Summary of RNA-seq for DSA, DSB, and DSC. [file Table_4.DOCX]

| **Table S4 Summary of RNA-seq for DSA, DSB and DSC** | | | | | | |
| --- | --- | --- | --- | --- | --- | --- |
| **Sample name** | **Raw reads** | **Clean reads** | **Clean bases** | **mapped resds** | **mapping ratio** | **GC content(%)** |
| DsA1 | 59969028 | 59519186 | 8.93G | 47498758 | 79.80% | 46.14 |
| DsA2 | 83783996 | 83168806 | 12.48G | 60771756 | 73.07% | 45.96 |
| DsA3 | 64718010 | 64064420 | 9.61G | 49548136 | 77.34% | 45.58 |
| DsB1 | 60768064 | 60077626 | 9.01G | 43827320 | 72.95% | 45.91 |
| DsB2 | 61522862 | 60899274 | 9.13G | 48781212 | 80.10% | 45.67 |
| DsB3 | 67426760 | 66601390 | 9.99G | 50980924 | 76.55% | 45.71 |
| DsC1 | 55199622 | 54481224 | 8.17G | 44115828 | 80.97% | 45.96 |
| DsC2 | 65358024 | 64653772 | 9.7G | 43968270 | 68.01% | 46.21 |
| DsC3 | 50010118 | 49688904 | 7.45G | 38304784 | 77.09% | 45.79 |
| Total | 568756484 | 563154602 | 84.47G | 427796988 | 75.96% | - |
